# Fast and Non-Destructive Profiling of Commercial Coffee Aroma under Three Conditions (Beans, Powder, and Brews) Using GC-IMS

**DOI:** 10.3390/molecules27196262

**Published:** 2022-09-23

**Authors:** Yanping Chen, He Chen, Dandan Cui, Xiaolei Fang, Jie Gao, Yuan Liu

**Affiliations:** Department of Food Science & Technology, School of Agriculture & Biology, Shanghai Jiao Tong University, Shanghai 200240, China

**Keywords:** coffee, E-nose, GC-IMS, volatile organic components

## Abstract

The flavor of coffee can be affected by the preparation parameters. In this investigation, the flavor profiles of three coffee brands under three conditions (bean, powder, and brew) were analyzed by gas chromatography—ion mobility spectrometry (GC-IMS) and the electronic nose (E-nose). The flavor results were further studied using multiple factor analysis (MFA). A total of 117 peaks were identified in all coffee samples by GC-IMS, and the principal component analysis (PCA) showed these coffee samples could be grouped and separated. A total of 37 volatile organic compounds (VOCs) were selected as biomarkers to distinguish coffee samples, including 5 aldehydes, 10 ketones, 8 alcohols, 2 acids, 4 esters, 5 furans, and 3 other compounds. The comparison between E-nose and GC-IMS data using partial least squares regression (PLSR) and MFA showed GC-IMS could present very close sample spaces. Compared with E-nose, GC–IMS could not only be used to classify coffee samples in a very short time but also provide VOC bio-markers to discriminate coffee samples.

## 1. Introduction

Coffee, originating from Ethiopia, has become one of the most popular drinks in the world. The typical coffee flavor is mainly caused by volatile organic compounds (VOCs) generated during the roasting process at a high temperature (above 200 °C), when flavor precursors inside raw coffee beans undergo the Maillard reaction, Strecker degradation, caramelization, and pyrolysis [1]. The attractive coffee flavors include nutty, caramel, floral, and chocolate, which might owe to aldehydes, ketones, alcohols, sulfur-containing compounds, and volatile heterocyclic compounds. The sulfur-containing compounds originate from the thermal degradation of sulfur-containing amino acids in the presence of sugars, and heterocyclic compounds, such as pyrazine, pyrrole, and pyridine, are yielded by the Strecker reaction between amino acids and aldehydes [2,3].

Coffee flavor can be identified by many analytical methods. The most commonly used techniques, such as solid-phase micro-extraction–gas chromatography–mass spectrometry (SPME-GC-MS), and gas chromatography–olfactometry (GC-O) can be used to predict volatile compounds (e.g., aldehydes and pyrazines) in single-roasted coffee beans [4]. In one study, ultra-performance liquid chromatography–time-of-flight mass spectrometry (UPLC-TOF-MS) revealed a marker substance for a coffee beverage spiked with acetaldehyde [5]. Flash gas chromatography electronic nose (FGC E-nose) can obtain a more convincing flavor assessment than a single assessment of aroma or volatile profiles [6].

The electronic nose (E-nose) is a novel instrument that imitates the human olfactory process to analyze and recognize complex odors efficiently. Previous studies have shown good discrimination of roasted coffee, instant coffee, and coffee products with different roasting degrees using E-nose [7]. However, compared to gas chromatography–mass spectrometry (GC-MS), E-nose can only respond to profiles from sensor arrays, and the volatiles contributing to the odors cannot be identified. Originating in the 1970s [8], ion mobility spectrometry (IMS) coupled with GC can be used to screen and distinguish food samples in a very short time. It contains a low detection limit at the trace level (ng/L) without pretreatment. A comparison between the principal component analysis (PCA) of GC-IMS and two-dimensional gas chromatography–mass spectrometry (GC×GC-MS) volatiles and multiple factor analysis (MFA) results showed that GC-IMS could be used as a fast way to discriminate dry-cured ham products in different regions in China [9]. The comparison between GC-IMS and ultra-fast GC E-nose showed they both could be used to discriminate between raw and cooked fish [10]. GC-IMS was used to identify the authenticity of Iberian ham samples and reported VOCs such as octan-2-one, trans-2-octenal, and nonan-2-one as biomarkers [11]. In addition, a total of 26 volatile metabolites (aldehydes, ketones, alcohols, and esters) were identified to facilitate the characterization of specific attributes of olive oils [12]. 

The flavor of coffee is affected by many parameters, such as natural environmental factors (light intensity, plant density, and rainfall) and the processing conditions (roasting temperature, and roasting time) [13]. The varied preparation methods of coffee brews also result in varied coffee flavors. The extract levels of flavor components depend on the ground particle size of roasted coffee beans and the temperature of hot water poured over coffee powder [3,14]. The cations contained inside coffee beans and dissolved inside water for coffee brew extraction may have an influence on the coffee aroma [15]. Therefore, the objectives of this study were (1) to distinguish commercial coffee under three conditions (bean, powder, and brew) using GC-IMS, (2) to identify potential biomarkers to separate those samples using GC-IMS, and (3) to compare the discrimination ability between GC-IMS and E-nose. This study could develop a fast method for the discrimination of coffee with varied flavors. 

## 2. Results and Discussion

### 2.1. GC-IMS Results

**Figure 1:**
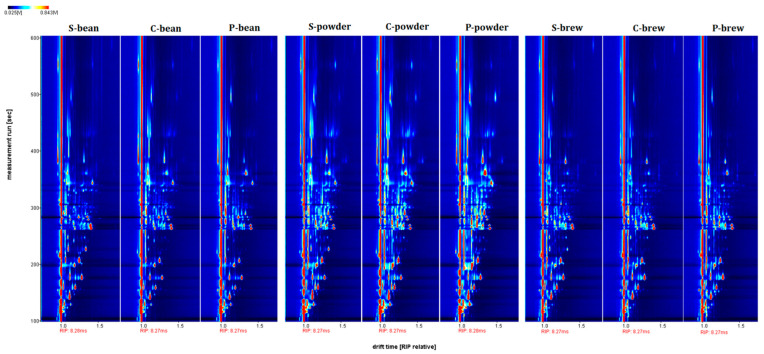
Fingerprints of VOCs isolated from three commercial coffee samples (S, C, and P) presented in bean, powder, and brew, respectively. The ordinate shows the retention time, and the abscissa shows the drift time. The background of the plot is blue, and the red vertical line on the left side represents the reactive ion peak (RIP, normalized drift time of 8.27 ms).

The VOCs isolated from the three commercial coffee samples (S, C, and P) in the bean, powder, and brew forms are shown in Figure 1. The Figure 1 showed the background is blue. The red vertical line at the abscissa, 1.0, is the reactive ion peak (RIP). The X-axis and Y-axis represent the drift time and retention time, respectively [16]. The signals were mostly located within the retention time range, from 100 to 400 s, and a drift time from 1.0 to 1.5 riprel. Each detected VOC produced a signal that presented as a colored spot. The concentration variances of each VOC depended on the signal intensity shown in the varied colors, with red representing higher intensity and blue meaning not present.

**Figure 2:**
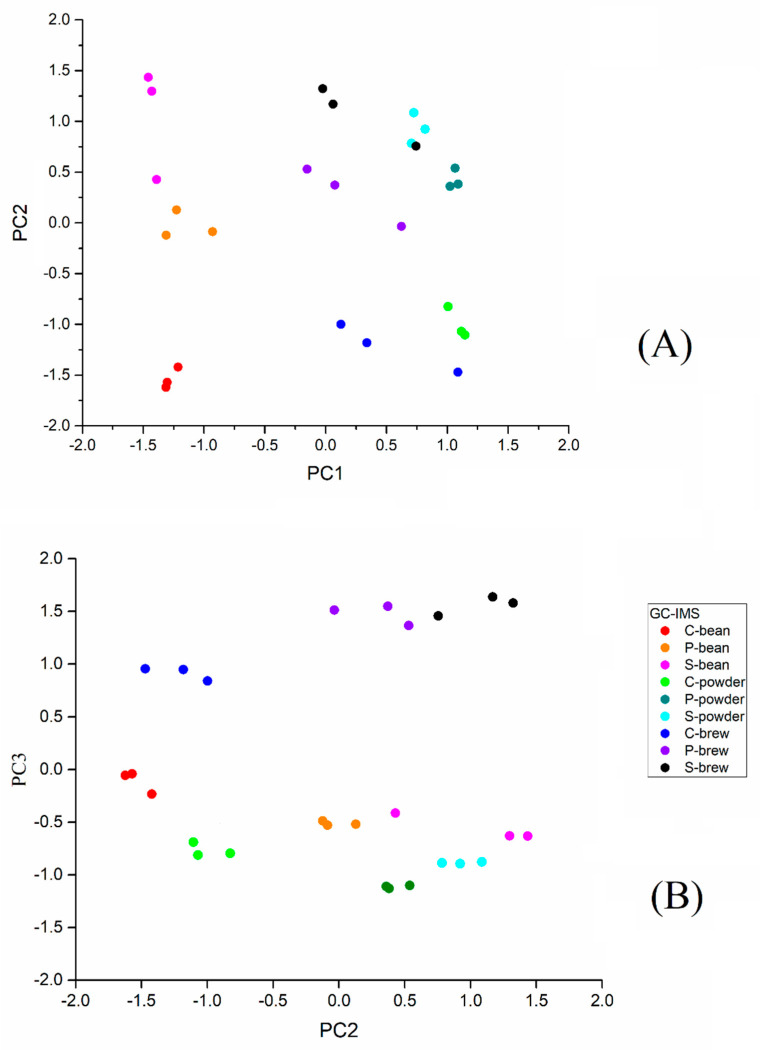
Principal component analysis of the flavor profiles of three commercial coffee samples (S, C, and P) in three conditions (bean, powder, and brew) obtained by GC−IMS on PC1−PC2 (**A**) and PC2−PC3 (**B**).

A total of 117 signals were detected in all samples (data not shown). To visualize the vast amount of data by reducing dimensions, PCA was performed based on the identified signals using GC-IMS. The PCA was a multivariate statistical analysis technique. Through dimensional reduction analysis, the selected PCs could represent the contribution rates in different samples [17]. Figure 2 shows the score plots obtained via PCA, and each dot represents a sample. The dots of the same color represent the replicates of the sample. The first and second principal components (PC1 and PC2) explained 45.8% (Figure 2A) variance and the second and third principal components (PC2 and PC3) explained 40.3% (Figure 2B) variance.

From the results (Figure 2A), it can be seen that three brands of coffee beans scattered along PC1 and PC2. The powder was located in a similar position along the positive side of PC1 and grouped into three categories along PC2. The coffee brew was mostly located on the positive side of PC1, and the coffee bean was located on the negative side of PC1. In Figure 2B, the coffee brew is located on the upper positive side of PC3, the coffee bean is located on the negative side of PC3 (−0.5 to 0), and the coffee powder is located on the negative downside of PC3 (−0.5 to −1.0). The distribution diagram shows that the different statuses of the coffee samples could be distinguished by PC1 and PC2 (Figure 2A) and PC2 and PC3 (Figure 2B), i.e., bean, powder, and brew. 

Figure 2B shows the coffee bean, powder, and brew of three brands (C, P, and S) to be separated in PC2. In Figure 2B, brand C is located more separately from brand P and brand S, including its bean, powder, and brew forms, indicating that the flavors of brand P and brand S are more similar. The results showed coffee samples could be distinguished by brands and status. This suggested that their flavor profiles were different from each other and could be distinguished by their brands. Figure 2 indicates that GC-IMS can differentiate coffee samples of different brands in bean, powder, and brew using PCA.

### 2.2. Biomarkers Identified Using GC-IMS

A total of 37 compounds were tentatively identified as important markers for discrimination, including 5 aldehydes, 10 ketones, 8 alcohols, 2 acids, 4 esters, 5 furans, and 3 other compounds (Table 1). The dimer eluted after the monomer, included butanal, butanone, 2-pentanone, ethanol, 2-ethylfuran, and furfural (Table 1), because the molecular mass of the dimer was higher than that of the monomer counterparts [18]. We found that butanal dimer, pentanal, 2-propanone, 2,3-butanedione, butanone monomer, butanone dimer, methyl isobutyl, 2-propanol, furfural dimer, and ethyl pyrazine had higher signal intensities (>60) in each sample. These compounds commonly existed in all three brands of coffee in the bean, powder, and brew forms and developed the typical coffee flavor. The 2,3-butanedione contributed a buttery, fruity, and caramel-like aroma [19,20]. Furfural was most likely generated by Amadori rearrangement through the 3-deoxyosone pathway or, alternatively, via the oxidation of furfuryl alcohol with the almond aroma [21]. Ethyl pyrazine contributed a roasted and nutty odor [22].

In addition, the signal intensities of butanal, 2-pentanone, and methyl acylate were higher in coffee S; the 3-pentanone and isoamyl acetate levels in coffee P were higher than in other samples; and the concentrations of furfural and 5-methylfurfural in C were higher than in S and P. The signal intensities of butanal monomer, butanal dimer, propanal, butanone monomer, 2-pentanone monomer, 2-pentanone dimer, 3-pentanone, butanol, 2-ethyl-1-propanol, 2-methyl-1-butanol, acetic acid, propanoic acid, methyl acrylate, athyl acetate monomer, 2-pentanone, 3-pentanone acetic acid, 2-ethylfuran, 2-methyl-1-butanol, methyl acrylate, propanal, and propane 2-methoxy-2-methyl- in S were higher than in P and C for the bean, powder, and brew statuses. The majority of hydrocarbon compounds were reported to have high odor thresholds, so they rarely contributed to coffee flavor. Acid compounds usually produce an unpleasant odor. Acetic acid is the volatile compound in coffee, having a sharp pungent odor like vinegar [19,23]. The concentration of acids will reduce in the process of roasting. 2-methyl-1-butanol was detected in all samples, which had a malt flavor. 2-methylpropanol was also found in all samples with almost the same number of concentrations. Furan could be generated by the thermal treatment of Maillard reaction precursors or lipids [24,25]. The formation of furan may also depend on the content of sugar [24]. 2-ethylfuran contributes a pungent and fruity flavor to coffee. Methyl pyrazine has a nutty, roasted, and chocolate aroma [26]. 

Isoamyl acetate, methional, and ethyl pyrazine in P had higher concentrations than in S and C. The methional in the bean and powder forms were significantly higher than in the brews. Methional is the key coffee odorant [5], which is generated by a Strecker reaction between α-dicarbonyl and the amino acid methionine. In addition, furfural, 5-methylfurfural, 3-methyl-2-butanol, 2,3-pentanedione, and 2-ethylfuran levels in C were higher than in S and P. 5-methylfurfural presents with a caramel, spicy, and maple flavor in coffee [19,27]. 2,3-pentanedione is considered one source of the buttery aroma in coffee [6,19], with an odor threshold of 30 ppb.

### 2.3. Comparison between GC-IMS and E-Nose

**Figure 3:**
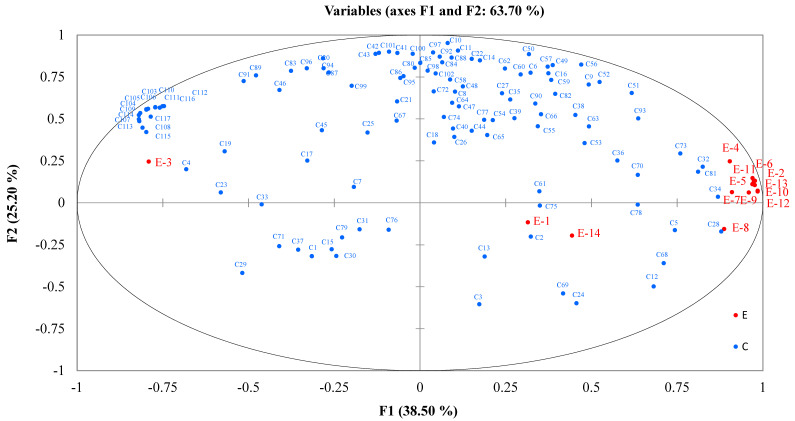
PLSR analysis to study the correlation between GC-IMS signals and E-nose sensor arrays. The VOCs identified by GC-IMS showed as C1 to C117 (blue dots). Data measured by E-nose showed as E1 to E 14 (red dots).

The flavor responses from the E-nose were based on signal responses from 14 sensors (Table S1). In order to further investigate the correlation between the E-nose and GC-IMS data, the PLSR was used for correlation analysis, where the GC-IMS data was used as the independent variable (X), and the E-nose data was used as the dependent variable (Y). The circle represents 100% of the explained variance. Among the variables explained by F1, most of the E-nose data (except E-1, E-3, and E-14) are located on the right side of the relevant load diagram, while the GC-IMS data are also located in the relevant location. As shown in Figure 3, the reference substance for the E-nose gas sensor array, E3 response to hydrogen, which had a strong positive correlation with the VOCs, such as C4, C115, and C108, measured by GC-IMS. The reference substances of sensors E1 and E14 were ammonia, amines, methane, and fuel gas and had a positive correlation with the VOCs, such as C2 and C75, measured by GC-IMS. Other sensing substances were mainly positively correlated with C28, C34, C81, and C32, located on the right side along the X-axis (Figure 3).

**Figure 4:**
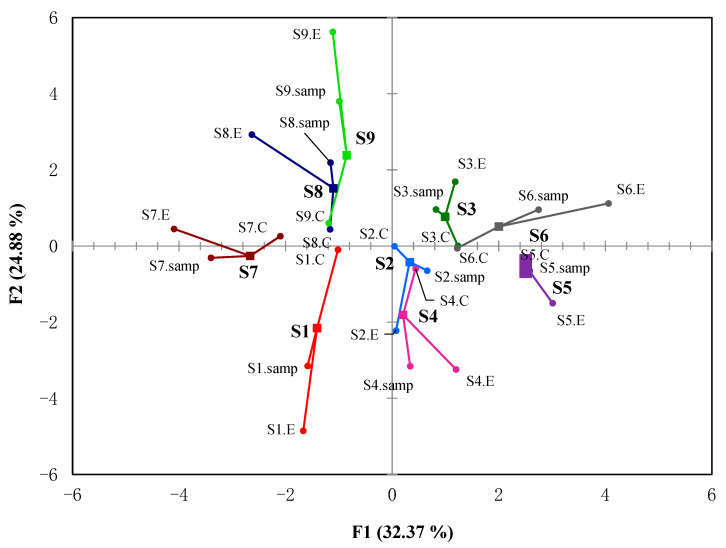
Comparison of the flavor profiles of three commercial coffee samples (S, C, and P) in three conditions (bean, powder, and brew) using multiple factor analysis (MFA). S1, S2, and S3 represent the flavor characteristics of three commercial coffee samples (S, C, and P) under bean conditions; S4, S5, and S6 represent the flavor characteristics under powder conditions; S7, S8, and S9 represent the flavor characteristics under brewing conditions.

The correlation between the sample statuses, E-nose, and GC-IMS was then analyzed using multiple factor analysis (MFA) (Figure 4), where F1 and F2 accounted for 63.7% of variances. MFA is useful in studying relationships by simultaneously analyzing observations, variables, and tables [28]. In Figure 4, the centroid represents the resulting coordinates of the MFA. The points connected to the centroid represent the coordinates of the projections formed by the variations. The closer these projections are to the centroid, the greater the similarity between the descriptions. In Figure 4, S1, S2, and S3 represent the flavor characteristics of three commercial coffee samples (S, C, and P) in bean conditions; S4, S5, and S6 represent the flavor characteristics in powder conditions; S7, S8, and S9 represent the flavor characteristics in brewing conditions. Taking the average value of the E-nose and GC-IMS data as the sample value, from each sample value, it can be concluded that the same coffee sample dispersed further under different conditions, indicating that different conditions have a greater impact on coffee flavor characteristics. However, under the same conditions, different coffees are more closely distributed, indicating that even if the coffee samples are different, their flavor characteristics are similar under the same conditions. As can be seen in Figure 4, sample status and GC-IMS variables had a close distance to the centroid, indicating that GC-IMS is a good choice to discriminate coffee samples under different statuses. Our investigation showed GC-IMS could be a fast way to distinguish different coffee samples just like the E-noses do. Since E-noses only collect responses from 14 sensor arrays, GC-IMS could provide more VOCs, which could be served as biomarkers to indicate flavor differences in detail. The traditional GC-MS identified more VOCs, but a GC-IMS could show biomarkers in a time-saving and intuitive way.

## 3. Materials and Methods

### 3.1. Coffee Samples

Three brands of medium roasted coffee beans (S, C, and P) were purchased from local retailers and stored in a vacuum seal. They were *Arabica* beans from Colombia with moderately baking. The coffee powder was prepared by grinding the coffee with a manual grinder then passing it through a 700 µm sieve. Beverage was prepared as follows: 5.5 g of coffee powder was weighted into a coffee press beaker, added to 100 mL 92 °C hot water for brewing, immediately divided into a 20 mL headspace bottle, and sealed.

### 3.2. Gas Chromatography–Ion Mobility Spectrometry (GC-IMS)

Analyses of different coffee samples were performed on a commercial GC-IMS instrument (FlavourSpec^®^, Gesellschaft für Analytische Sensorsysteme, Dortmund, Germany) at Zhejiang University. The GC was connected to an Rtx-wax capillary column (30 m × 0.32 mm, 0.25 µm) and equipped with an auto-sampler unit (CTC Analytics AG, Zwingen, Switzerland), which used a 1 mL, air-tight, heated syringe to directly inject sample into the headspace.

The sample analysis method can be found in our previous report [16]. Samples (solid: 0.3 g, liquid: 1 mL) were transferred into a 20 mL vial and incubated at 40 °C for 20 min. Then, 100 µL headspace was automatically injected into the heated injector (80 °C, splitless mode) using a heated syringe (65 °C) at a speed of 500 µL/s. The carrier gas (Nitrogen, purity 99.99%) transferred the headspace of samples into GC column initially at a rate of 2 mL/min for 4 min, increased to 80 mL/min in 6 min, and, lastly, increased to 150 mL/min in 10 min. The analytes were eluted and separated at 45 °C then driven into an ionization chamber and ionized by a 3H ionization source with 300 MBq activity in positive ion mode. The resulting ions were driven to a drift tube (9.8 cm in length), which was operated at a constant temperature (45 °C) and voltage (5 kV). The flow rate of the drift gas (nitrogen gas) was set at 150 mL/min. Each spectrum was reported as the average of 12 scans. 

The retention index (RI) of each compound was calculated by using C4–C9 n-ketone mixture standards (Sinopharm Chemical Reagent Co., Ltd., Beijing, China) as external references. Volatile compounds were identified based on RI and drift time compared with the GC-IMS library supplied by G.A.S. (Gesellschaft für analytische Sensor system mbH). The volatile compounds identified from IMS data were realized by the coupled software Laboratory Analytical Viewer (LAV, version 2.0.0), including three plug-ins and a GC-IMS library search to analyze samples from different perspectives. 

### 3.3. E-Nose

The E-nose (Super Nose, Isenso Group Cooperation, Shanghai, China) used in this study was equipped with an array of 14 sensors. Each sensor was sensitive to hydrogen sulfide, sulfide, alcohols, ketones, aldehydes, and aromatic compounds.

The detection method can be found in our previous report [29]. Samples (solid: 3 g, liquid: 10 mL) were measured in a 100 mL beaker, covered tightly with a sealing film, and left at room temperature (25 °C) for 45 min until they reached equilibrium. Then, 20 mL of the headspace was extracted using a plastic, odorless free needle and injected into the sensor array chamber via sampling tubing. The sampling gas flowed at a constant speed rate of 600 mL/min and lasted for 160 s. The cleaning phase proceeded by pumping clean air for 100 s to normalize sensor signals between samples. Each sample replicated 12 parallels.

### 3.4. Statistical Analysis

The significant differences between coffee samples were found using SPSS statistical 23.0 software (IBM Watson Analytics, Beijing, China) at a *p* = 0.05 level with Duncan’s multiple comparison method. PCA was analyzed using OriginPro 2016 (Northampton, MA, USA). Multiple factor analysis (MFA) was analyzed using XLSTAT 2016 (New York, NY, USA).

## 4. Conclusions

In this study, 117 peaks were identified in three coffee brands under three conditions (bean, powder, and brew) by GC-IMS. The PCA results clearly showed that three different brands of coffee under the same treatment could be divided into three parts and could be distinguished well using GC-IMS. In addition, 37 signal peaks from topographic plots were selected as biomarkers for the identification and classification of coffee samples. The comparison between E-nose and GC-IMS data using PLSR and MFA showed GC-IMS could present very close sample spaces. Compared with E-nose, GC–IMS could not only be used to classify coffee samples in a very short time but also provide VOC biomarkers to discriminate coffee samples.

## Figures and Tables

**Table 1:** Compounds in three brands (P, S, C) in coffee bean, powder, and brew identified by GC-IMS.

No.	Compounds	CAS	RI	Dt/ Riprel	C-Bean	P-Bean	S-Bean	C-Powder	P-Powder	S-Powder	C-Brew	P-Brew	S-Brew
	**Aldehydes (5)**												
1	butanal monomer	123-72-8	546	1.3	21 ± 2 ^a^	23 ± 3 ^a^	96 ± 6 ^d^	27 ± 3 ^ab^	23 ± 2 ^ab^	94 ± 6 ^d^	46 ± 4 ^c^	30 ± 3 ^b^	98 ± 3 ^d^
2	butanal dimer	123-72-8	615	1.1	86 ± 5 ^b^	80 ± 7 ^ab^	93 ± 9 ^c^	98 ± 2 ^c^	83 ± 0 ^b^	75 ± 1 ^a^	99 ± 1 ^c^	96 ± 1 ^c^	94 ± 1 ^c^
3	pentanal	110-62-3	660	1.2	85 ± 7 ^bc^	95 ± 7 ^c^	84 ± 21 ^bc^	43 ± 5 ^a^	63 ± 3 ^ab^	90 ± 8 ^c^	81 ± 19 ^bc^	87 ± 16 ^c^	85 ± 12 ^bc^
4	propanal	123-38-6	805	1.1	11 ± 2 ^a^	56 ± 5 ^c^	91 ± 15 ^e^	35 ± 3 ^b^	79 ± 2 ^d^	96 ± 6 ^e^	15 ± 3 ^a^	62 ± 4 ^c^	97 ± 5 ^e^
5	methional	3268-49-3	916	1.1	52 ± 6 ^b^	91 ± 11 ^de^	77 ± 1 ^cd^	71 ± 1 ^c^	94 ± 3 ^de^	99 ± 2 ^e^	33 ± 10 ^a^	44 ± 21 ^ab^	50 ± 13 ^ab^
	**Ketones (10)**												
6	2-propanone	67-64-1	500	1.1	85 ± 1 ^a^	92 ± 7 ^bc^	88 ± 3 ^ab^	99 ± 0 ^d^	99 ± 1 ^d^	98 ± 0 ^cd^	95 ± 1 ^cd^	98 ± 0 ^cd^	99 ± 1 ^d^
7	2,3-butanedione	431-03-8	571	1.2	91 ± 4 ^bc^	94 ± 6 ^bc^	80 ± 15 ^b^	97 ± 3 ^c^	86 ± 2 ^bc^	60 ± 5 ^a^	94 ± 6 ^bc^	85 ± 9 ^bc^	80 ± 11 ^b^
8	butanone monomer	78-93-3	590	1.1	62 ± 5 ^a^	83 ± 15 ^bc^	83 ± 14 ^bc^	95 ± 7 ^c^	85 ± 9 ^c^	65 ± 8 ^ab^	78 ± 12 ^abc^	89 ± 9 ^c^	93 ± 8 ^c^
9	butanone dimer	78-93-3	587	1.1	80 ± 5 ^a^	80 ± 9 ^a^	92 ± 7 ^bcd^	94 ± 2 ^cd^	97 ± 3 ^d^	89 ± 1 ^bc^	86 ± 1 ^ab^	95 ± 0 ^cd^	99 ± 1 ^d^
10	2,3-pentanedione	600-14-6	671	1.3	90 ± 10 ^de^	66 ± 5 ^c^	25 ± 5 ^a^	99 ± 1 ^e^	89 ± 2 ^de^	77 ± 4 ^cd^	41 ± 11 ^b^	33 ± 11 ^ab^	28 ± 9 ^a^
11	2-pentanone monomer	107-87-9	686	1.1	23 ± 6 ^a^	66 ± 7 ^cd^	86 ± 19 ^e^	40 ± 5 ^b^	72 ± 3 ^d^	92 ± 7 ^e^	55 ± 1 ^c^	87 ± 1 ^e^	98 ± 2 ^e^
12	2-pentanone dimer	107-87-9	685	1.4	40 ± 1 ^a^	74 ± 2 ^bc^	90 ± 14 ^cd^	75 ± 3 ^bc^	97 ± 4 ^d^	78 ± 2 ^bcd^	60 ± 16 ^b^	83 ± 16 ^cd^	86 ± 15 ^cd^
13	3-pentanone	96-22-0	693	1.4	36 ± 9 ^a^	78 ± 6 ^b^	86 ± 20 ^bc^	88 ± 3 ^bc^	97 ± 4 ^c^	74 ± 2 ^b^	74 ± 1 ^b^	96 ± 1 ^c^	98 ± 2 ^c^
14	3-hydroxy-2-butanone	513-86-0	701	1.3	93 ± 6 ^cd^	91 ± 8 ^cd^	60 ± 1 ^b^	98 ± 2 ^d^	91 ± 1 ^cd^	84 ± 2 ^c^	15 ± 7 ^a^	14 ± 7 ^a^	15 ± 7 ^a^
15	methyl isobutyl	108-10-1	723	1.2	63 ± 2 ^a^	97 ± 2 ^d^	95 ± 4 ^d^	71 ± 9 ^abc^	93 ± 3 ^d^	94 ± 6 ^d^	69 ± 13 ^ab^	86 ± 14 ^cd^	81 ± 13 ^bcd^
	**Alcohols (8)**												
16	ethanol monomer	64-17-5	482	1.0	99 ± 1 ^ef^	95 ± 3 ^def^	92 ± 6 ^d^	99 ± 1 ^f^	93 ± 2 ^de^	84 ± 1 ^c^	94 ± 6 ^def^	48 ± 3 ^a^	54 ± 3 ^b^
17	ethanol dimer	64-17-5	482	1.1	81 ± 4 ^c^	51 ± 4 ^b^	44 ± 3 ^a^	90 ± 2 ^d^	92 ± 3 ^d^	97 ± 3 ^e^	99 ± 1 ^e^	80 ± 3 ^c^	87 ± 2 ^d^
18	isopropyl alcohol	67-63-0	506	1.2	52 ± 6 ^bc^	77 ± 26 ^cd^	68 ± 16 ^c^	97 ± 4 ^e^	40 ± 16 ^ab^	28 ± 11 ^a^	95 ± 5 ^de^	60 ± 5 ^bc^	65 ± 1 ^c^
19	2-propanol	123-38-6	546	1.3	58 ± 2 ^a^	80 ± 18 ^bc^	74 ± 9 ^b^	92 ± 1 ^cd^	98 ± 2 ^d^	93 ± 1 ^d^	87 ± 0 ^cd^	97 ± 1 ^d^	99 ± 1 ^d^
20	butanol	71-36-3	660	1.2	25 ± 1 ^a^	28 ± 5 ^a^	49 ± 1 ^b^	82 ± 15 ^cd^	71 ± 4 ^c^	53 ± 2 ^b^	92 ± 9 ^d^	82 ± 5 ^cd^	94 ± 3 ^d^
21	2-methyl-1-propanol	78-83-1	673	1.2	77 ± 6 ^b^	83 ± 10 ^b^	91 ± 9 ^b^	96 ± 3 ^b^	73 ± 5 ^b^	40 ± 9 ^a^	86 ± 9 ^b^	77 ± 28 ^b^	80 ± 21 ^b^
22	3-methyl-2-butanol	598-75-4	692	1.2	98 ± 3 ^e^	82 ± 3 ^bc^	68 ± 2 ^a^	98 ± 2 ^e^	93 ± 2 ^de^	89 ± 1 ^cd^	94 ± 8 ^de^	76 ± 7 ^b^	64 ± 6 ^a^
23	2-methyl-1-butanol	137-32-6	776	1.5	21 ± 7 ^a^	67 ± 12 ^b^	97 ± 5 ^c^	84 ± 14 ^c^	95 ± 2 ^c^	64 ± 10 ^b^	50 ± 6 ^b^	92 ± 9 ^c^	86 ± 10 ^c^
	**Acids (2)**												
24	acetic acid	64-19-7	635	1.2	40 ± 5 ^a^	74 ± 4 ^b^	90 ± 9 ^c^	72 ± 8 ^b^	92 ± 6 ^c^	93 ± 8 ^c^	65 ± 8 ^b^	87 ± 8 ^c^	95 ± 8 ^c^
25	propanoic acid	79-09-4	694	1.1	48 ± 3 ^a^	77 ± 2 ^c^	91 ± 10 ^d^	45 ± 3 ^a^	73 ± 3 ^bc^	94 ± 6 ^d^	61 ± 10 ^b^	90 ± 11 ^d^	91 ± 9 ^d^
	**Esters (4)**												
26	methyl acrylate	96-33-3	575	1.3	25 ± 3 ^a^	61 ± 23 ^bc^	98 ± 3 ^e^	36 ± 3 ^a^	72 ± 12 ^cd^	87 ± 11 ^de^	42 ± 12 ^ab^	63 ± 10 ^bc^	92 ± 10 ^de^
27	ethyl acetate monomer	141-78-6	641	1.1	39 ± 4 ^a^	79 ± 15 ^cd^	88 ± 15 ^de^	53 ± 6 ^ab^	89 ± 8 ^de^	95 ± 5 ^de^	65 ± 6 ^bc^	89 ± 8 ^de^	96 ± 5 ^e^
28	isoamyl acetate	123-92-2	854	1.3	38 ± 14 ^abc^	77 ± 25 ^de^	54 ± 3 ^bcd^	92 ± 6 ^e^	98 ± 2 ^e^	74 ± 4 ^de^	13 ± 6 ^a^	22 ± 8 ^ab^	37 ± 13 ^cd^
29	ethyl acetate dimer	141-78-6	868	1.1	68 ± 9 ^c^	92 ± 9 ^ef^	74 ± 6 ^cd^	94 ± 2 ^ef^	98 ± 2 ^f^	85 ± 2 ^de^	21 ± 4 ^a^	28 ± 5 ^ab^	38 ± 10 ^b^
	**Furans (5)**												
30	2-ethylfuran monomer	3208-16-0	681	1.1	44 ± 6 ^b^	62 ± 10 ^c^	88 ± 12 ^de^	98 ± 3 ^e^	22 ± 3 ^a^	31 ± 2 ^a^	92 ± 9 ^de^	85 ± 3 ^de^	84 ± 4 ^d^
31	2-ethylfuran dimer	3208-16-0	681	1.1	77 ± 3 ^bc^	73 ± 10 ^b^	84 ± 15 ^bc^	70 ± 1 ^b^	90 ± 4 ^bc^	95 ± 5 ^c^	14 ± 0 ^a^	26 ± 11 ^a^	80 ± 23 ^bc^
32	furfural monomer	98-01-1	823	1.3	93 ± 8 ^f^	41 ± 8 ^d^	13 ± 1 ^a^	98 ± 2 ^f^	50 ± 3 ^e^	31 ± 1 ^c^	99 ± 1 ^f^	31 ± 2 ^c^	22 ± 1 ^b^
33	furfural dimer	98-01-1	825	1.1	92 ± 8 ^de^	85 ± 5 ^cd^	59 ± 4 ^a^	64 ± 3 ^a^	87 ± 4 ^cd^	96 ± 4 ^e^	97 ± 3 ^e^	83 ± 2 ^c^	72 ± 2 ^b^
34	5-methylfurfural	620-02-0	967	1.1	90 ± 10 ^c^	71 ± 7 ^b^	39 ± 2 ^a^	99 ± 1 ^c^	73 ± 2 ^b^	45 ± 1 ^a^	94 ± 6 ^c^	64 ± 10 ^b^	51 ± 9 ^a^
	**Others (3)**												
35	dimethyl sulfide	75-18-3	517	1.0	50 ± 3 ^c^	23 ± 7 ^a^	29 ± 7 ^ab^	92 ± 7 ^e^	34 ± 1 ^b^	21 ± 1 ^a^	98 ± 2 ^e^	64 ± 1 ^d^	47 ± 1 ^c^
36	propane 2-methoxy-2-methyl	96-33-3	575	1.3	50 ± 3 ^a^	76 ± 9 ^bc^	99 ± 1 ^d^	84 ± 4 ^cd^	94 ± 6 ^cd^	51 ± 6 ^a^	58 ± 16 ^ab^	79 ± 15 ^c^	89 ± 13 ^cd^
37	ethyl pyrazine	13925-00-3	911	1.1	92 ± 9 ^d^	91 ± 1 ^d^	74 ± 6 ^bc^	98 ± 2 ^d^	67 ± 1 ^ab^	61 ± 3 ^a^	99 ± 1 ^d^	79 ± 3 ^c^	74 ± 4 ^bc^

Rt, retention time. Dt, drift time. Different letters in the same row indicate statistical differences between samples at a *p* = 0.05 level.

## Data Availability

The data presented in this study are available on request from the corresponding author.

## Supplementary Materials

The following supporting information can be downloaded at: https://www.mdpi.com/article/10.3390/molecules27196262/s1, Table S1: The flavor responses from the E-nose based on signal responses from 14 sensors.

## Sample Availability

Samples of the compounds are available or not from the authors.
