# Supplementary material for: Fast and Non-Destructive Profiling of Commercial Coffee Aroma under Three Conditions (Beans, Powder, and Brews) Using GC-IMS
Source: Molecules. 2022 Sep 23;27(19):6262. doi: 10.3390/molecules27196262 (PMC9572980; doi:10.3390/molecules27196262)
Supplement: Supplementary file 1 [file molecules-27-06262-s001.zip › molecules-1905483-Supplementary.pdf]

**Table S1.** The flavor responses from the E-nose based on signal responses from 14 sensors.

| Samples | E-1 | E-2 | E-3 | E-4 | E-5 | E-6 | E-7 | E-8 | E-9 | E-10 | E-11 | E-12 | E-13 | E-14 |
|---|---|---|---|---|---|---|---|---|---|---|---|---|---|---|
| S1 | 0.348 | 6.445 | 0.360 | 3.588 | 6.604 | 1.437 | 0.768 | 24.179 | 2.800 | 2.096 | 6.271 | 7.032 | 5.608 | 2.808 |
| S2 | 0.212 | 10.926 | 0.301 | 3.928 | 10.750 | 2.053 | 1.010 | 38.237 | 3.009 | 2.425 | 10.261 | 7.605 | 6.009 | 2.532 |
| S3 | 0.338 | 14.920 | 0.403 | 4.860 | 15.423 | 2.354 | 1.067 | 63.047 | 3.815 | 3.264 | 15.314 | 9.689 | 7.680 | 2.097 |
| S4 | 0.313 | 15.933 | 0.290 | 4.682 | 15.938 | 2.215 | 0.911 | 50.908 | 3.353 | 2.995 | 16.689 | 9.200 | 7.210 | 2.098 |
| S5 | 0.335 | 19.836 | 0.293 | 7.755 | 19.263 | 3.033 | 1.307 | 46.495 | 4.821 | 4.216 | 20.649 | 12.362 | 10.694 | 3.856 |
| S6 | 0.269 | 16.673 | 0.332 | 5.671 | 16.381 | 2.293 | 1.008 | 45.974 | 3.777 | 3.237 | 17.142 | 10.010 | 8.247 | 2.802 |
| S7 | 0.247 | 5.157 | 0.542 | 3.379 | 4.993 | 1.253 | 0.646 | 8.362 | 2.532 | 1.583 | 5.016 | 5.244 | 4.120 | 3.188 |
| S8 | 0.296 | 5.371 | 0.467 | 3.602 | 5.308 | 1.581 | 0.897 | 12.261 | 2.449 | 1.806 | 5.078 | 6.256 | 5.104 | 1.733 |
| S9 | 0.280 | 6.077 | 0.431 | 3.470 | 5.822 | 1.397 | 0.703 | 13.533 | 2.349 | 1.691 | 5.769 | 5.911 | 4.724 | 1.496 |
